# *N*′-(2,4-Dichloro­benzyl­idene)-2-hydr­oxy-3-methyl­benzohydrazide

**DOI:** 10.1107/S1600536810012419

**Published:** 2010-04-10

**Authors:** You-Yue Han, Qiu-Rong Zhao

**Affiliations:** a Department of Chemistry and Life Sciences, Chuzhou University, Chuzhou, Anhui 239000, People’s Republic of China

## Abstract

In the title compound, C_15_H_12_Cl_2_N_2_O_2_, the dihedral angle between the two benzene rings is 6.3 (2)°. The mol­ecule adopts an *E* configuration with respect to the C=N bond. An intra­molecular O—H⋯O hydrogen bond is observed. In the crystal structure, the mol­ecules are linked through inter­molecular N—H⋯O and C—H⋯O hydrogen bonds to form chains running along [101].

## Related literature

For the biological properties of hydrazone compounds, see: Patil *et al.* (2010 ▶); Cukurovali *et al.* (2006 ▶). For related structures, see: Mohd Lair *et al.* (2009 ▶); Lin & Sang (2009 ▶); Suleiman Gwaram *et al.* (2010 ▶); Li & Ban (2009 ▶); Lo & Ng (2009 ▶); Ning & Xu (2009 ▶); Zhu *et al.* (2009 ▶). For bond-length data, see: Allen *et al.* (1987 ▶).
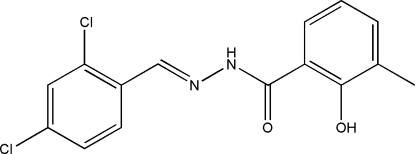


## Experimental

### 

#### Crystal data

C_15_H_12_Cl_2_N_2_O_2_
*M* *_r_* = 323.17
Monoclinic, 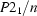

*a* = 7.137 (1) Å
*b* = 28.146 (2) Å
*c* = 8.130 (1) Å
β = 115.098 (1)°
*V* = 1478.9 (3) Å^3^
*Z* = 4
Mo *K*α radiation
μ = 0.44 mm^−1^
*T* = 298 K
0.20 × 0.20 × 0.17 mm

#### Data collection

Bruker SMART CCD area-detector diffractometer
Absorption correction: multi-scan (*SADABS*; Bruker, 2001 ▶) *T*_min_ = 0.917, *T*_max_ = 0.928
8543 measured reflections
3211 independent reflections
2439 reflections with *I* > 2σ(*I*)
*R*_int_ = 0.080

#### Refinement

*R*[*F* ^2^ > 2σ(*F* ^2^)] = 0.045
*wR*(*F* ^2^) = 0.130
*S* = 1.08
3211 reflections
195 parameters
1 restraint
H atoms treated by a mixture of independent and constrained refinement
Δρ_max_ = 0.20 e Å^−3^
Δρ_min_ = −0.34 e Å^−3^

### 

Data collection: *SMART* (Bruker, 2007 ▶); cell refinement: *SAINT* (Bruker, 2007 ▶); data reduction: *SAINT*; program(s) used to solve structure: *SHELXTL* (Sheldrick, 2008 ▶); program(s) used to refine structure: *SHELXTL*; molecular graphics: *SHELXTL*; software used to prepare material for publication: *SHELXTL*.

## Supplementary Material

Crystal structure: contains datablocks global, I. DOI: 10.1107/S1600536810012419/ci5073sup1.cif

Structure factors: contains datablocks I. DOI: 10.1107/S1600536810012419/ci5073Isup2.hkl

Additional supplementary materials:  crystallographic information; 3D view; checkCIF report

## Figures and Tables

**Table 1:** Hydrogen-bond geometry (Å, °)

*D*—H⋯*A*	*D*—H	H⋯*A*	*D*⋯*A*	*D*—H⋯*A*
O1—H1⋯O2	0.82	1.96	2.6689 (19)	144
N2—H2⋯O2^i^	0.90 (1)	2.13 (1)	2.9905 (19)	161 (2)
C7—H7⋯O2^i^	0.93	2.45	3.264 (2)	146

Symmetry code: (i) 
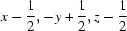
.

**Fig. 1.**
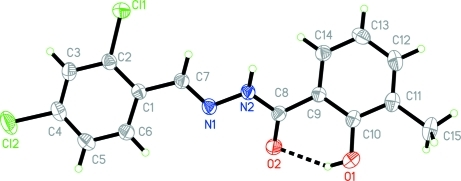
The molecular structure of the title compound with atom labels and 30% probability displacement ellipsoids for non-H atoms. An intramolecular hydrogen bond is shown as a dashed line.

**Fig. 2.**
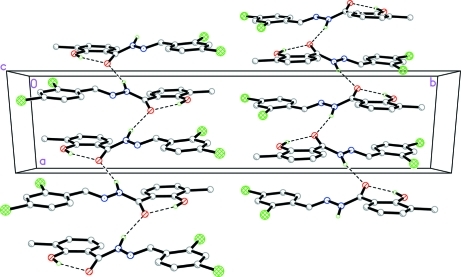
The molecular packing of the title compound, viewed along the c axis. Hydrogen bonds are shown as dashed lines.

## supplementary crystallographic information

### Comment

Hydrazone compounds have been widely investigated for their biological properties (Patil *et al.*, 2010; Cukurovali *et al.*, 2006). Furthermore, the crystal structures of the hydrazone compounds have also attracted much attention in recent years (Mohd Lair *et al.*, 2009; Lin & Sang, 2009; Suleiman Gwaram *et al.*, 2010). In the present work, the title new hydrazone compound is reported.

In the title molecule (Fig. 1), the dihedral angle between the two benzene rings is 6.3 (2)°. The molecule adopts an *E* configuration with respect to the C═N bond. There is an intramolecular O—H···O hydrogen bond (Table 1) in the molecule. All the bond lengths are within normal ranges (Allen *et al.*, 1987), and are comparable to those observed in related structures (Li & Ban, 2009; Lo & Ng, 2009; Ning & Xu, 2009; Zhu *et al.*, 2009).

In the crystal structure, molecules are linked through intermolecular N—H···O and C—H···O hydrogen bonds (Table 1) to form chains running along the [101] (Fig. 2).

### Experimental

A mixture of 2,4-dichlorobenzaldehyde (0.174 g, 1 mmol) and 2-hydroxy-3-methylbenzohydrazide (0.166 g, 1 mmol) in methanol (50 ml) was stirred at room temperature for 1 h. The mixture was filtered to remove impurities, and then left at room temperature. After a few days, single crystals of the title compound, suitable for X-ray diffraction, were formed.

### Refinement

Atom H2 was located in a difference Fourier map and refined isotropically, with the N–H distance restrained to 0.90 (1) Å. Other H atoms were positioned geometrically and refined using the riding-model approximation, with C–H = 0.93 or 0.96 Å, O–H = 0.82 Å, and U_iso_(H) = 1.2U_eq_(C) or U_iso_(H) = 1.5U_eq_(methyl C and O).

### Crystal data

**Table:**

C_15_H_12_Cl_2_N_2_O_2_	*F*(000) = 664
*M**_r_* = 323.17	*D*_x_ = 1.451 Mg m^−^^3^
Monoclinic, *P*2_1_/*n*	Mo *K*α radiation, λ = 0.71073 Å
Hall symbol: -P 2yn	Cell parameters from 3312 reflections
*a* = 7.137 (1) Å	θ = 2.7–26.7°
*b* = 28.146 (2) Å	µ = 0.44 mm^−^^1^
*c* = 8.130 (1) Å	*T* = 298 K
β = 115.098 (1)°	Block, colourless
*V* = 1478.9 (3) Å^3^	0.20 × 0.20 × 0.17 mm
*Z* = 4	

### Data collection

**Table:**

Bruker SMART CCD area-detector diffractometer	3211 independent reflections
Radiation source: fine-focus sealed tube	2439 reflections with *I* > 2σ(*I*)
graphite	*R*_int_ = 0.080
ω scans	θ_max_ = 27.0°, θ_min_ = 1.5°
Absorption correction: multi-scan (*SADABS*; Bruker, 2001)	*h* = −7→9
*T*_min_ = 0.917, *T*_max_ = 0.928	*k* = −35→29
8543 measured reflections	*l* = −10→10

### Refinement

**Table:**

Refinement on *F*^2^	Primary atom site location: structure-invariant direct methods
Least-squares matrix: full	Secondary atom site location: difference Fourier map
*R*[*F*^2^ > 2σ(*F*^2^)] = 0.045	Hydrogen site location: inferred from neighbouring sites
*wR*(*F*^2^) = 0.130	H atoms treated by a mixture of independent and constrained refinement
*S* = 1.08	*w* = 1/[σ^2^(*F*_o_^2^) + (0.0634*P*)^2^ + 0.0199*P*] where *P* = (*F*_o_^2^ + 2*F*_c_^2^)/3
3211 reflections	(Δ/σ)_max_ = 0.001
195 parameters	Δρ_max_ = 0.20 e Å^−^^3^
1 restraint	Δρ_min_ = −0.34 e Å^−^^3^

### Special details

**Table:**

Geometry. All esds (except the esd in the dihedral angle between two l.s. planes) are estimated using the full covariance matrix. The cell esds are taken into account individually in the estimation of esds in distances, angles and torsion angles; correlations between esds in cell parameters are only used when they are defined by crystal symmetry. An approximate (isotropic) treatment of cell esds is used for estimating esds involving l.s. planes.
Refinement. Refinement of *F*^2^ against ALL reflections. The weighted *R*-factor wR and goodness of fit *S* are based on *F*^2^, conventional *R*-factors *R* are based on *F*, with *F* set to zero for negative *F*^2^. The threshold expression of *F*^2^ > σ(*F*^2^) is used only for calculating *R*-factors(gt) etc. and is not relevant to the choice of reflections for refinement. *R*-factors based on *F*^2^ are statistically about twice as large as those based on *F*, and *R*- factors based on ALL data will be even larger.

### Fractional atomic coordinates and isotropic or equivalent isotropic displacement parameters (Å^2^)

**Table:**

	*x*	*y*	*z*	*U*_iso_*/*U*_eq_	
Cl1	0.08171 (10)	0.072034 (18)	0.35298 (7)	0.0675 (2)	
Cl2	0.28271 (12)	0.02969 (2)	1.04608 (9)	0.0896 (3)	
N1	0.2165 (2)	0.21754 (5)	0.51447 (19)	0.0456 (3)	
N2	0.1732 (2)	0.24652 (5)	0.36590 (19)	0.0461 (4)	
O1	0.2584 (2)	0.39071 (4)	0.38436 (19)	0.0607 (4)	
H1	0.3024	0.3723	0.4706	0.091*	
O2	0.3710 (2)	0.30580 (5)	0.54178 (17)	0.0612 (4)	
C1	0.1840 (3)	0.13994 (6)	0.6149 (2)	0.0418 (4)	
C2	0.1588 (3)	0.09173 (6)	0.5740 (2)	0.0456 (4)	
C3	0.1937 (3)	0.05770 (7)	0.7075 (3)	0.0532 (5)	
H3	0.1815	0.0255	0.6790	0.064*	
C4	0.2466 (3)	0.07250 (7)	0.8822 (3)	0.0564 (5)	
C5	0.2684 (3)	0.11985 (7)	0.9281 (3)	0.0571 (5)	
H5	0.3025	0.1293	1.0470	0.068*	
C6	0.2390 (3)	0.15312 (7)	0.7953 (2)	0.0507 (4)	
H6	0.2562	0.1851	0.8263	0.061*	
C7	0.1512 (3)	0.17531 (6)	0.4737 (2)	0.0447 (4)	
H7	0.0814	0.1667	0.3522	0.054*	
C8	0.2616 (3)	0.28989 (6)	0.3889 (2)	0.0440 (4)	
C9	0.2212 (2)	0.31687 (6)	0.2219 (2)	0.0412 (4)	
C10	0.2190 (3)	0.36649 (6)	0.2284 (2)	0.0458 (4)	
C11	0.1737 (3)	0.39357 (7)	0.0725 (3)	0.0530 (5)	
C12	0.1414 (3)	0.36996 (7)	−0.0855 (3)	0.0598 (5)	
H12	0.1124	0.3876	−0.1902	0.072*	
C13	0.1503 (3)	0.32088 (8)	−0.0946 (3)	0.0595 (5)	
H13	0.1306	0.3060	−0.2028	0.071*	
C14	0.1888 (3)	0.29443 (6)	0.0593 (2)	0.0487 (4)	
H14	0.1932	0.2615	0.0544	0.058*	
C15	0.1625 (4)	0.44689 (7)	0.0806 (4)	0.0719 (6)	
H15A	0.1534	0.4603	−0.0312	0.108*	
H15B	0.2845	0.4586	0.1797	0.108*	
H15C	0.0425	0.4558	0.0983	0.108*	
H2	0.077 (3)	0.2377 (8)	0.2565 (18)	0.080*	

### Atomic displacement parameters (Å^2^)

**Table:**

	*U*^11^	*U*^22^	*U*^33^	*U*^12^	*U*^13^	*U*^23^
Cl1	0.1002 (5)	0.0532 (3)	0.0537 (3)	−0.0007 (3)	0.0369 (3)	−0.0057 (2)
Cl2	0.1246 (6)	0.0848 (5)	0.0756 (4)	0.0201 (4)	0.0580 (4)	0.0368 (3)
N1	0.0454 (8)	0.0445 (8)	0.0405 (7)	−0.0003 (6)	0.0122 (6)	0.0052 (6)
N2	0.0480 (9)	0.0424 (8)	0.0379 (7)	−0.0048 (6)	0.0083 (6)	0.0040 (6)
O1	0.0731 (10)	0.0438 (7)	0.0648 (9)	−0.0015 (6)	0.0290 (8)	−0.0077 (6)
O2	0.0725 (9)	0.0499 (8)	0.0422 (7)	−0.0099 (6)	0.0060 (7)	−0.0029 (6)
C1	0.0380 (9)	0.0467 (9)	0.0401 (9)	0.0001 (7)	0.0158 (7)	0.0036 (7)
C2	0.0472 (10)	0.0483 (10)	0.0472 (9)	0.0026 (7)	0.0256 (8)	0.0032 (7)
C3	0.0599 (12)	0.0453 (10)	0.0631 (12)	0.0056 (8)	0.0344 (10)	0.0104 (9)
C4	0.0585 (12)	0.0638 (13)	0.0545 (11)	0.0116 (9)	0.0312 (10)	0.0189 (9)
C5	0.0597 (12)	0.0695 (13)	0.0416 (9)	0.0060 (9)	0.0211 (9)	0.0056 (9)
C6	0.0516 (11)	0.0515 (11)	0.0451 (10)	−0.0009 (8)	0.0168 (9)	0.0011 (8)
C7	0.0437 (10)	0.0457 (10)	0.0409 (9)	−0.0019 (7)	0.0143 (8)	0.0015 (7)
C8	0.0411 (9)	0.0408 (9)	0.0436 (9)	0.0018 (7)	0.0118 (8)	0.0015 (7)
C9	0.0346 (8)	0.0412 (9)	0.0425 (9)	−0.0015 (6)	0.0111 (7)	0.0013 (7)
C10	0.0369 (9)	0.0419 (9)	0.0550 (11)	0.0006 (7)	0.0159 (8)	0.0032 (8)
C11	0.0398 (10)	0.0487 (10)	0.0666 (12)	0.0021 (7)	0.0188 (9)	0.0103 (9)
C12	0.0507 (12)	0.0641 (13)	0.0593 (12)	−0.0033 (9)	0.0181 (10)	0.0198 (10)
C13	0.0579 (12)	0.0746 (14)	0.0435 (10)	−0.0112 (10)	0.0191 (9)	−0.0023 (9)
C14	0.0467 (10)	0.0469 (10)	0.0489 (10)	−0.0041 (8)	0.0167 (8)	−0.0011 (8)
C15	0.0662 (14)	0.0479 (12)	0.0980 (17)	0.0069 (9)	0.0313 (13)	0.0206 (11)

### Geometric parameters (Å, °)

**Table:**

Cl1—C2	1.7319 (18)	C5—H5	0.93
Cl2—C4	1.7323 (18)	C6—H6	0.93
N1—C7	1.268 (2)	C7—H7	0.93
N1—N2	1.3795 (19)	C8—C9	1.474 (2)
N2—C8	1.350 (2)	C9—C14	1.393 (2)
N2—H2	0.898 (10)	C9—C10	1.398 (2)
O1—C10	1.360 (2)	C10—C11	1.394 (3)
O1—H1	0.82	C11—C12	1.376 (3)
O2—C8	1.2376 (19)	C11—C15	1.506 (3)
C1—C2	1.391 (3)	C12—C13	1.386 (3)
C1—C6	1.398 (2)	C12—H12	0.93
C1—C7	1.461 (2)	C13—C14	1.379 (3)
C2—C3	1.389 (2)	C13—H13	0.93
C3—C4	1.371 (3)	C14—H14	0.93
C3—H3	0.93	C15—H15A	0.96
C4—C5	1.375 (3)	C15—H15B	0.96
C5—C6	1.376 (3)	C15—H15C	0.96
			
C7—N1—N2	113.86 (14)	O2—C8—C9	122.17 (16)
C8—N2—N1	119.69 (14)	N2—C8—C9	116.17 (14)
C8—N2—H2	120.2 (15)	C14—C9—C10	119.26 (16)
N1—N2—H2	119.8 (15)	C14—C9—C8	121.98 (16)
C10—O1—H1	109.5	C10—C9—C8	118.75 (15)
C2—C1—C6	117.41 (16)	O1—C10—C11	116.74 (17)
C2—C1—C7	121.03 (15)	O1—C10—C9	122.35 (16)
C6—C1—C7	121.56 (16)	C11—C10—C9	120.91 (17)
C3—C2—C1	121.58 (17)	C12—C11—C10	117.83 (17)
C3—C2—Cl1	117.58 (14)	C12—C11—C15	122.08 (19)
C1—C2—Cl1	120.84 (13)	C10—C11—C15	120.09 (19)
C4—C3—C2	118.68 (18)	C11—C12—C13	122.54 (18)
C4—C3—H3	120.7	C11—C12—H12	118.7
C2—C3—H3	120.7	C13—C12—H12	118.7
C3—C4—C5	121.67 (17)	C14—C13—C12	119.04 (19)
C3—C4—Cl2	118.12 (16)	C14—C13—H13	120.5
C5—C4—Cl2	120.20 (15)	C12—C13—H13	120.5
C4—C5—C6	119.02 (18)	C13—C14—C9	120.33 (18)
C4—C5—H5	120.5	C13—C14—H14	119.8
C6—C5—H5	120.5	C9—C14—H14	119.8
C5—C6—C1	121.58 (17)	C11—C15—H15A	109.5
C5—C6—H6	119.2	C11—C15—H15B	109.5
C1—C6—H6	119.2	H15A—C15—H15B	109.5
N1—C7—C1	120.98 (16)	C11—C15—H15C	109.5
N1—C7—H7	119.5	H15A—C15—H15C	109.5
C1—C7—H7	119.5	H15B—C15—H15C	109.5
O2—C8—N2	121.66 (16)		

### Hydrogen-bond geometry (Å, °)

**Table:**

*D*—H···*A*	*D*—H	H···*A*	*D*···*A*	*D*—H···*A*
O1—H1···O2	0.82	1.96	2.6689 (19)	144
N2—H2···O2^i^	0.90 (1)	2.13 (1)	2.9905 (19)	161 (2)
C7—H7···O2^i^	0.93	2.45	3.264 (2)	146

Symmetry codes: (i) *x*−1/2, −*y*+1/2, *z*−1/2.
